# Study on the aggregation patterns of fleas parasitizing the great gerbil (*Rhombomys opimus*) in the Junggar Basin plague natural focus

**DOI:** 10.1186/s13071-025-06676-4

**Published:** 2025-02-13

**Authors:** Fang Li, Guoyu Zhao, Yu Wang, Shang Zhan, Xine Tang, Tao Luo, Abulimiti Moming, Huiqian Wang, Jianhui Chen, Qiguo Wang, Haiyan Wu, Yujiang Zhang

**Affiliations:** 1 Department of Public Health, College of Xinjiang Medicine University, Ürümqi, Xinjiang Uygur Autonomous Region People’s Republic of China; 2 Key Laboratory of Vector-Borne Infectious Diseases, Ürümqi, Xinjiang Uygur Autonomous Region People’s Republic of China; 3 The First Affiliated Hospital of Xinjiang Medical University, Ürümqi, Xinjiang Uygur Autonomous Region People’s Republic of China; 4 Center for Disease Control and Prevention , Wenquan, Xinjiang Uygur Autonomous Region People’s Republic of China; 5 Center for Disease Control and Prevention, Ürümqi, Xinjiang Uygur Autonomous Region People’s Republic of China

**Keywords:** Aggregation, Fleas, Great gerbils, Taylor’s power law

## Abstract

**Background:**

The great gerbil (*Rhombomys opimus*), whose ectoparasitic fleas significantly influence the transmission and prevalence of plague, was the dominant rodent species in the Junggar Basin in Northwestern China. However, the distribution pattern of fleas parasitizing the great gerbils and whether that pattern affected the intensity of plague prevalence in different regions remains unclear.

**Methods:**

A total of 17,780 fleas were collected from 2258 great gerbils throughout 90 investigations. This study focused on analyzing the rate of flea infestation and the flea indices of species that parasitized the great gerbils. The aggregation patterns of fleas parasitizing the great gerbils were measured using the parameter *b* of Taylor’s power law, and the differences in the aggregation index of plague epidemic areas were compared.

**Results:**

We observed an aggregated distribution of ectoparasitic fleas in the great gerbils. The aggregation degree of combined fleas was higher (*P* > 0.05) in the eastern area of the Junggar Basin than in the western area. The primary species of ectoparasitic fleas of the great gerbils were *Xenopsylla skrjabini*, *Xenopsylla minax*, *Xenopsylla hirtipes*, and *Nosopsyllus laeviceps laiveceps*. *X. skrjabini* exhibited the highest (*P* < 0.01) degree of aggregation in the eastern zone (III), with an aggregation index of 1.61. In addition, in the middle zone (II), the aggregation index of *X. minax* and *X. hirtipes* reached their peak, with values of 1.53 and 1.56, respectively. Conversely, the degree of aggregation of *N. laeviceps* was more pronounced in the eastern zone than in the western zone of the Junggar Basin. Notably, the aggregation degree of the combined fleas of the great gerbils during the low-intensity plague epidemic period, with an index of 1.93, was significantly higher (*P* < 0.001) than during the high-intensity epidemic period, with an index of 1.50.

**Conclusions:**

Fleas exhibited an aggregated distribution within the great gerbil population. The levels of flea aggregation varied across zones characterized by differing intensities of plague epidemics. In addition, the degree of flea aggregation was significantly correlated with the intensity of plague prevalence.

**Graphical Abstract:**

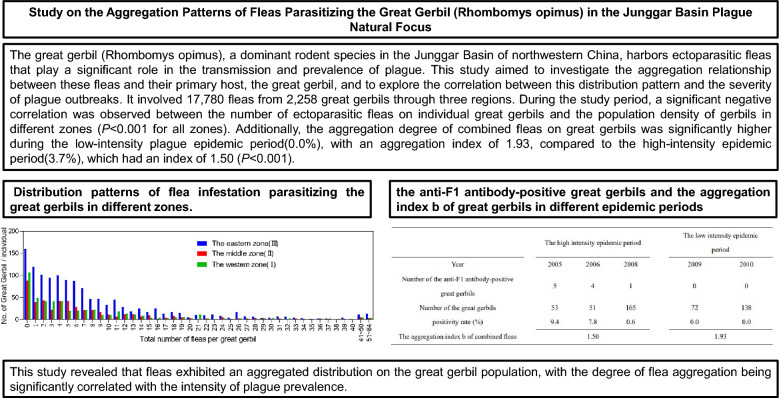

**Supplementary Information:**

The online version contains supplementary material available at 10.1186/s13071-025-06676-4.

## Background

Plague, caused by the bacterium *Yersinia pestis*, is a deadly flea-borne zoonotic disease [1, 2]. Fleas, the primary vector of plague, not only harbor *Y. pestis* but also transmit the bacteria to both host animals and humans through bites, thereby facilitating the spread of the disease [1, 3]. The investigation of the ecological and epidemiological aspects of fleas, along with their interactions with their hosts, has long been a primary area of interest in the study of plague. Studies have indicated that fleas exhibit a highly aggregated distribution on their hosts, whereby a limited number of host individuals harbor a substantial population of fleas, while the majority possess either few or none at all [4–6]. This aggregative distribution pattern significantly influences the stability of host-parasite populations [7–9]. Low levels of aggregation are frequently associated with instability in these dynamics, whereas high levels contribute to increased stability [7]. Furthermore, research has indicated that this distribution pattern affects the mating probabilities of parasites, the density-dependent regulation of both host and parasite populations, and competitive interactions among various parasite species [10]. However, the majority of studies have focused primarily on elucidating the factors responsible for parasite aggregation [11–14], while few studies have investigated the correlation between this distribution pattern and the severity of disease outbreaks.

In 2005, a newly discovered plague focus was identified in the Junggar Basin (Xinjiang, China) [15], with a natural habitat area of approximately 330,000 square kilometers and an altitude between 200 m and 500 m. The great gerbil has been identified in previous research as the primary host of the plague reservoir. The prevalence of plague among animals in this foci is closely associated with the community structure of ectoparasitic fleas found on the great gerbils [16]. Concurrently, long-term monitoring data have indicated that the epidemic has exhibited significant spatial heterogeneity and temporal fluctuations, suggesting a complex and dynamic process [17]. Currently, the distribution pattern of fleas parasitizing the great gerbils and whether that pattern affects the prevalence of plague in the foci remains unclear. Consequently, this study aimed to employ Taylor’s power law to investigate the aggregation relationship between fleas and their primary host, the great gerbil, from the perspective of varying intensities of animal plague epidemics in different regions.

## Methods

### Data sources

The data for this study were derived from continuous and comprehensive surveillance of fleas parasitizing individuals of the great gerbil in the Junggar Basin focus, provided by the Xinjiang Center for Disease Control and Prevention. This dataset covers the period from 2005 to 2010 and has been publicly published.

### Study area

On the basis of the plague antibody positivity rate of the great gerbil in the Junggar Basin, the focus was divided into three subregions: the western zone (I), the middle zone (II), and the eastern zone (III) of the Junggar Basin. Among them, the western zone (I) of the Junggar Basin includes Wusu, Jinghe, and Tole counties. The middle zone (II), located in the western section of the Gurbantunggut Desert within the eastern part of the Junggar Basin, includes Karamay and Hefeng counties. The eastern zone (III), located in the middle-eastern section of the Gurbantunggut Desert in the eastern part of the Junggar Basin, includes Changji, Fukang, Jimsar, Qitai, Mulei, Hutubi, Manasi, and Buerjin counties.

### Statistical analyses

For each trapping survey of the great gerbil, a series of calculations were performed to determine the infestation rate, flea index, and their variance. In addition, the base-10 logarithms of both the flea index and its variance were computed for each flea species identified. The flea index (a measure of fleas per host) [18] was calculated by dividing the number of fleas collected from the great gerbils by the total number of great gerbils [19]. All calculations were conducted via Microsoft Excel software version 16.0. Furthermore, a Chi-squared test was employed to ascertain whether there were disparities in the flea infestation rates in various zones. The Kruskal–Wallis *H* test was applied to assess potential fluctuations in the flea index in these zones. When noteworthy differences were identified, the Dunnett test was subsequently implemented for a more granular comparison among the groups.

Taylor’s power law [20], expressed as ${V}_{m}=a\times {m}^{b}$, is a mathematical model used to quantify the degree of aggregation in parasitic fleas. This law is often transformed into a linear regression model in logarithmic form ${\text{log}}_{10}\left({V}_{m}\right)={\text{log}}_{10}a+{\text{b}\times \text{log}}_{10}m$ to facilitate calculation [21]. Moreover, a bootstrapping technique [22] was employed to calculate the parameter *b* of Taylor’s power law, ensuring robustness against outliers and enabling accurate estimation of *b* for each subsample in the analyzed data. Specifically, the program randomly sampled (with replacement) 50 flea counts and calculated the ${\text{log}}_{10}m$ and ${\text{log}}_{10}\left({V}_{m}\right)$ of each subsample. This process was repeated 100 times with an estimate of *b* calculated as the slope from the linear regression of ${\text{log}}_{10}m$ onto ${\text{log}}_{10}\left({V}_{m}\right)$.To ensure the reliability of the regression model and the statistical interpretability of the parameters, we performed normality and heteroscedasticity tests on the residuals of the regression models. The significance level for all the statistical tests was set at 0.05, and all the analyses were performed using R version 4.1.3.

## Results

### Flea composition parasitizing the great gerbil

A total of 90 flea investigations were conducted on the great gerbils in three subzones. Consequently, a total of 2258 great gerbils were captured, from which 17,780 fleas belonging to 19 species of 10 genera and 8 families were collected. The mean flea infestation rate of the great gerbils was 83.7% [standard error (SE) =  ± 0.5], and the mean total flea index was 7.95 (SE =  ±1.72). Notably, the average numbers of flea species *X. skrjabini*, *X. minax*, *X. hirtipes*, and *N. laeviceps* accounted for more than 5% of the total average flea count, with percentages of 35.3%, 33.3%, 9.3%, and 6.0%, respectively, indicating their status as common ectoparasites of great gerbils (Additional File 1, Table S1).

### Differences in the composition of primary ectoparasitic fleas of great gerbils in different zones

A total of 14 flea investigations were conducted on great gerbils in the western zone (I) of the Junggar Basin, resulting in the capture of 492 great gerbils and the collection of 3369 fleas belonging to 15 species. The mean flea infestation rate and total flea index were 71.0% (SE =  ±6.9) and 6.32 (SE =  ±1.14), respectively. In the middle zone (II) and the eastern zone (III), a total of 18 and 58 flea investigations were conducted, resulting in the capture of 478 and 1288 great gerbils, respectively. The mean flea infestation rate and total flea index for the two zones were 83.2% (SE =  ±3.3), 7.35 (SE =  ±0.87), 86.9% (SE =  ±1.6), and 8.52 (SE =  ±0.74), respectively (Table 1).

**Table 1:** Parasitological parameters of the primary species of ectoparasitic fleas on the great gerbils in the three zones

Zones	Subzones	Symbol	No. of surveys	No. of great gerbils	Flea infestation rate % (±SE)	Total flea index (±SE)	Indices of primary species of ectoparasitic fleas			
							*X. skrjabini*	*X. minax*	*X. hirtipes*	*N. laeviceps*
West of the Junggar Basin (Clay Desert)	The western zone	I	14	492	71.0 (6.9)	6.32 (1.14)	0.84	3.75	0.26	0.62
East of the Junggar Basin (Gurbantungut Desert)	The middle zone	II	18	478	83.2 (3.3)	7.35 (0.87)	1.98	3.01	0.38	0.44
	The eastern zone	III	58	1288	86.9 (1.6)	8.52 (0.74)	5.66	0.10	1.31	0.42
	Summary		90	2258	83.7 (0.5)	7.95 (1.72)	3.83	1.51	0.88	0.47

SE, standard error

Furthermore, the flea infestation rate exhibited a significant disparity across the three zones (*χ*^2^ = 25.49, *df* = 2, *P* < 0.001), with an increasing trend from the western to the eastern parts of the Junggar Basin. The flea infestation rate of the great gerbils in the western zone (I) was the lowest 71.0% (SE =  ±6.9), which was lower than in the middle zone (II) 83.2% (±3.3). However, the difference between the two zones was not statistically significant (*χ*^2^ = 1.70, *df* = 1, *P* = 0.192). In addition, the flea infestation rate in the western zone (I) was significantly lower (*χ*^2^ = 23.31, *df* = 1, *P* < 0.001) than in the eastern zone (III), with a rate of 86.9% (SE =  ± 1.6). Furthermore, a statistically significant difference in the flea infestation rate was found between the middle zone (II) and the eastern zone (III) (*χ*^2^ = 99.68, *df* = 1, *P* = 0.002).

There was no significant difference observed in the total flea index across the three zones (*H* = 1.35, *df* = 2, *P* = 0.509) or in the flea index of *N. laeviceps* (*H* = 1.80, *df* = 2, *P* = 0.406). However, there were statistically significant differences in the flea indices of *X. skrjabini*, *X. minax*, and *X. hirtipes* in the three areas (*H*_*X. skrjabini*_ = 25.68, *df* = 2, *P*_*X. skrjabini*_ < 0.001; *H*_*X. minax*_ = 29.17, *df* = 2, *P*_*X. minax*_ < 0.001; *H*_*X. hirtipes*_ = 14.28, *df* = 2, *P*_*X. hirtipes*_ < 0.001).

Further analysis via the Dunnett test revealed significant differences in the flea indices among the three species in the zones. The indices for *X. skrjabini* and *X. hirtipes* increased from west to east in the Junggar Basin (*X. skrjabini*: *H*_I–III_ = −4.41, *df* = 1, *P*_I–III_ < 0.001; *H*_II–III_ = −3.38, *df* = 1, *P*_II–III_ = 0.001; *X. hirtipes: H*_I–III_ = −2.84, *df* = 1, *P*_I–III_ = 0.007; *H*_II, III_ = −3.03, *df* = 1, *P*_II, III_ = 0.004). Conversely, the index for *X. minax* showed a decreasing trend from west to east in the Junggar Basin, with statistically significant differences in the three areas (*H*_I–III_ = 4.33, *df* = 1, *P*_I–III_ < 0.001; *H*_II, III_ = 3.93, *df* = 1, *P*_II, III_ < 0.001).

### Distribution of the major parasitic fleas on individual great gerbils

Across all zones, the great gerbils harbored one to two species of fleas. Notably, infested individuals accounted for 84.2% of the total gerbil population in the three zones, with the highest proportion observed in the eastern zone (III) at 87.5% (Table 2). The dominant flea species harbored by great gerbils varies geographically in terms of their ability to host a single species of flea. For instance, the western zone (I) and the middle zone (II) were inhabited primarily by *X. minax*, accounting for 66.0% and 49.0%, respectively. Conversely, the eastern zone (III) was inhabited mainly by *X. skrjabini*, accounting for 84.5% of the total area. In terms of coinfection with two species of fleas, the composition of parasitic flea associations varied across zones. The western zone (I) was predominantly characterized by coinfections of *X. minax* and *N. laeviceps*, accounting for 56.7%. In contrast, the middle zone (II) primarily exhibited coinfections of *X. skrjabini* and *N. laeviceps*, accounting for 47.8%, whereas the eastern zone (III) was characterized by coinfections of *X. skrjabini* and *X. hirtipes*, accounting for 73.1%. Moreover, in terms of the number of flea species infesting the gerbils, the eastern zone (III) had the highest number of infected flea species, with six species, whereas each of the other two zones harbored five species (Table 2).

**Table 2:** Distribution of flea species parasitizing the great gerbils in different zones

No. of flea species	The western zone (I)		The middle zone (II)		The eastern zone (III)	
	No. of great gerbils	Percentage (%)	No. of great gerbils	Percentage (%)	No. of great gerbils	Percentage (%)
0	106	21.5	88	18.4	160	12.4
1	161	32.7	173	36.2	407	31.6
2	156	31.7	108	22.6	482	37.0
3	48	9.8	86	18.0	195	15.0
4	16	3.3	20	4.2	41	3.2
5	5	1.0	3	0.6	9	0.7
6	0	0.0	0	0.0	1	0.1

**Fig. 1:**
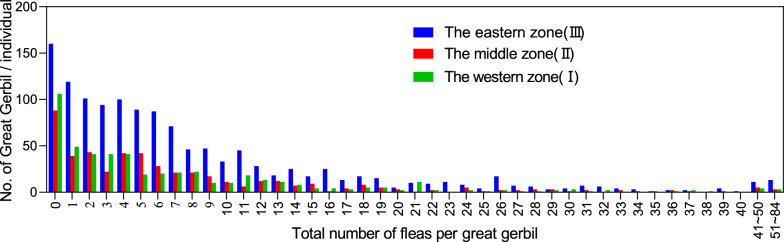
Distribution patterns of flea infestation parasitizing the great gerbils in different zones

The number of fleas on individual great gerbils ranged from 0 to 84 across the three zones, with the highest proportion being gerbils free of parasitic fleas, accounting for 15.8%. In addition, there was a significant negative correlation between the number of ectoparasitic fleas on individual great gerbils and the number of gerbils in different zones (*rs*_I_ = −0.85, *P*_I_ < 0.001; *rs*_II_ = −0.83, *P*_II_ < 0.001; *rs*_III_ = −0.91, *P*_III_ < 0.001), indicating that within the great gerbil population, the majority of individuals carried only a small number of fleas, whereas a minority had a higher aggregation of ectoparasitic fleas (Fig. 1).

To further elucidate the distribution pattern of flea populations on great gerbils infested with ectoparasitic fleas, we categorized the number of fleas into 15 groups and subsequently calculated the level of flea infestation for each zone and group. In addition, we determined the flea infestation ratio and flea collection ratio of infected gerbils, as presented in Table 3. With respect to the abundance of parasitic fleas, a majority of great gerbils were found to harbor between one and five fleas, constituting 46.3% of the total population. However, the groups encompassing six to ten fleas and 11 to 15 fleas made the most significant contributions to the overall flea index for great gerbils, collectively accounting for 36.3% of the entire flea collection.

**Table 3:** Distribution of flea quantities parasitizing the great gerbils in different zones

Groups	The western zone (I)			The middle zone (II)			The eastern zone (III)		
	No. of infested great gerbils	Percentage^a^ (%)	Percentage^b^ (%)	No. of infested great gerbils	Percentage^a^ (%)	Percentage^b^ (%)	No. of infested great gerbils	Percentage^a^ (%)	Percentage^b^ (%)
0	106	21.5	0.0	88	18.4	0.0	160	12.4	0.0
1–5	191	38.8	15.2	188	39.3	16.0	503	39.1	13.4
6–10	83	16.9	18.8	98	20.5	20.9	284	22.0	19.7
11–15	54	11.0	19.9	46	9.6	16.8	133	10.3	15.4
16–20	19	3.9	10.1	21	4.4	10.7	75	5.8	12.1
21–25	16	3.3	10.3	8	1.7	5.3	42	3.3	8.8
26–30	9	1.8	7.6	11	2.3	8.6	37	2.9	9.3
31–35	3	0.6	2.8	6	1.3	5.5	21	1.6	6.3
36–40	4	0.8	4.4	4	0.8	4.2	9	0.7	3.2
41–45	3	0.6	3.7	5	1.1	6.0	8	0.6	3.1
46–50	1	0.2	1.5	0	0.0	0.0	3	0.2	1.4
51–55	1	0.2	1.6	1	0.2	1.5	5	0.4	2.5
56–60	1	0.2	1.8	0	0.0	0.0	1	0.1	0.5
61–65	0	0.0	0.0	0	0.0	0.0	2	0.2	1.2
66–70	0	0.0	0.0	0	0.0	0.0	4	0.3	2.5
71–84	1	0.2	2.4	2	0.4	4.5	1	0.1	0.8

^a^Percentage of infected great gerbils out of the total population (%)

^b^Percentage of fleas out of the total population (%)

### Analysis of the aggregation of the primary ectoparasitic fleas on the great gerbil

#### Model evaluation

All regression models were statistically significant (*P* < 0.05), indicating a logarithmic linear relationship between the flea index variance and the flea index itself. Furthermore, the residuals of the regression models were normally distributed, and no evidence of heteroscedasticity was found, suggesting that this model effectively explained the variability in the distributions of different ectoparasitic fleas among the great gerbil populations (Table 4).

**Table 4:** Linear regression estimates of the parameters of Taylor’s law ${\text{log}}_{10}\left({V}_{m}\right)=a+b\times {\text{log}}_{10}m$, with *m* = flea index, ${V}_{m}$= variance of the relative flea index collected in different regions

Zone	Flea species	Intercept_a (± SE)	Aggregation index_b (± SE)	Adj. *R*^2^	*df*	Swilk	Heteroskedasticity
The western zone (I)	Combined fleas	0.30 (0.19)	1.79 (0.23)	0.83	11	ns	ns
	*X. skrjabini*	0.69 (0.09)	1.42 (0.15)	0.93	6	ns	ns
	*X. minax*	0.82 (0.17)	1.21 (0.21)	0.80	7	ns	ns
	*X. hirtipes*	0.76 (0.18)	1.26 (0.18)	0.93	3	ns	ns
	*N. laeviceps*	0.37 (0.08)	1.20 (0.15)	0.86	10	ns	ns
The middle zone (II)	Combined fleas	0.22 (0.22)	1.82 (0.26)	0.74	16	ns	ns
	*X. skrjabini*	0.62 (0.06)	1.42 (0.07)	0.97	11	ns	ns
	*X. minax*	0.55 (0.08)	1.53 (0.12)	0.95	8	ns	ns
	*X. hirtipes*	0.57 (0.13)	1.56 (0.18)	0.92	5	ns	ns
	*N. laeviceps*	0.50 (0.07)	1.35 (0.11)	0.91	14	ns	ns
The eastern zone (III)	Combined fleas	0.19 (0.09)	1.70 (0.13)	0.82	56	ns	ns
	*X. skrjabini*	0.36 (0.06)	1.61 (0.08)	0.89	56	ns	ns
	*X. minax*	0.63 (0.12)	1.47 (0.18)	0.93	4	ns	ns
	*X. hirtipes*	0.46 (0.03)	1.31 (0.05)	0.94	41	ns	ns
	*N. laeviceps*	0.39 (0.05)	1.37 (0.07)	0.90	43	ns	n

Intercept_a, the least-squares estimate of the intercept *a* of Taylor’s law; aggregation index_b, the least-squares estimate of the slope *b* of Taylor’s law; Adj. *R*^2^, adjusted *R*^2^ (adjusted for the number of fit parameters); *df*, error (residual) degrees of freedom (*df*) equals number of observations minus the number of fitted parameters; Swilk, Shapiro–Wilk test of the normality of residuals from the linear regression of Taylor’s law;

SE, standard error; ns, not significant at the 5% level;

heteroskedasticity, *P*-value of the test of the null hypothesis that the residuals from the linear regression are homoskedastic, i.e., all have the same variance

The aggregation index *b* for the combined fleas and the four primary flea species exceeded 1, indicating an aggregated distribution of ectoparasitic fleas on the great gerbils (Table 4). The differences in the aggregation index for the combined fleas showed no statistically significant variation across the three subzones (*H* = 5.76, *df* = 2, *P* = 0.056).

### Analysis of the aggregation patterns of the main parasitic fleas in different zones

The level of aggregation varied among the four primary parasitic fleas in three zones (*H*_*X. skrjabini*_ = 129.79, *df* = 2, *P*_*X. skrjabini*_ < 0.001; *H*_*X. minax*_ = 143.50, *df* = 2, *P*_*X. minax*_ < 0.001; *H*_*X. hirtipes*_ = 161.31, *P*_*X. hirtipes*_ < 0.001; *H*_*N. laeviceps*_ = 64.34, *df* = 2, *P*_*N. laeviceps*_ < 0.001). The results of the Dunnett test further demonstrated that the aggregation index for *X. skrjabini* was highest in the eastern zone (III), at 1.61. Similarly, *X. minax* and *X. hirtipes* presented the highest aggregation indices in the middle zone (II), at 1.53 and 1.56, respectively, both of which were significantly higher than those found in the western zone (I). Furthermore, *N. laeviceps* showed a higher degree of aggregation in the eastern zone than in the western zone of the Junggar Basin (Table 5).

**Table 5:** Comparison of the levels of aggregation among four primary flea species on great gerbils in different zones

Flea species	The western zone (I) versus the middle zone (II)		The western zone (I) versus the eastern zone (III)		The middle zone (II) versus the eastern zone (III)	
	*H*-value	*P*-value	*H*-value	*P*-value	*H*-value	*P*-value
*X. skrjabini*	1.65	> 0.05	−8.94	< 0.01**	−10.59	< 0.01**
*X. minax*	−10.28	< 0.01**	−10.46	< 0.01**	−0.21	> 0.05
*X. hirtipes*	−11.68	< 0.01**	−1.51	> 0.05	10.17	< 0.01**
*N. laeviceps*	−6.08	< 0.01**	−7.57	< 0.01**	−1.49	> 0.05

^**^Statistically significant

### Analysis of the aggregation of fleas parasitizing the great gerbils during different periods of plague prevalence intensity

On the basis of the seroprevalence of *Y. pestis* antibodies in great gerbils, the animal plague prevalence in Alashankou, located within the western zone (I) of the Junggar Basin from 2005 to 2010, was divided into two distinct stages with varying intensities. The high-intensity epidemic period was from 2005 to 2008, with a positive rate of 3.7% (10/269), whereas the low-intensity epidemic period was from 2009 to 2010, with a positive rate of 0.0% (0/210) (Table 6). Our study revealed that the aggregation degree of the combined fleas on the great gerbils during the low-intensity prevalence period (*b* = 1.93) was significantly higher than during the high-intensity prevalence period (*b* = 1.50), with a statistically significant difference (*H* = 59.12, *df* = 1, *P* < 0.001) (Table 6).

**Table 6:** Results of anti-F1 antibody-positive great gerbils and the aggregation index *b* of great gerbils in different epidemic periods in Alashankou, located within the western zone (I)

	The high-intensity epidemic period			The low-intensity epidemic period	
Year	2005	2006	2008	2009	2010
Number of the anti-F1 antibody-positive great gerbils	5	4	1	0	0
Number of great gerbils	53	51	165	72	138
Positivity rate (%)	9.4	7.8	0.6	0.0	0.0
The aggregation index *b* of combined fleas	1.50			1.93	

### Analysis of aggregation patterns among different flea species

**Fig. 2:**
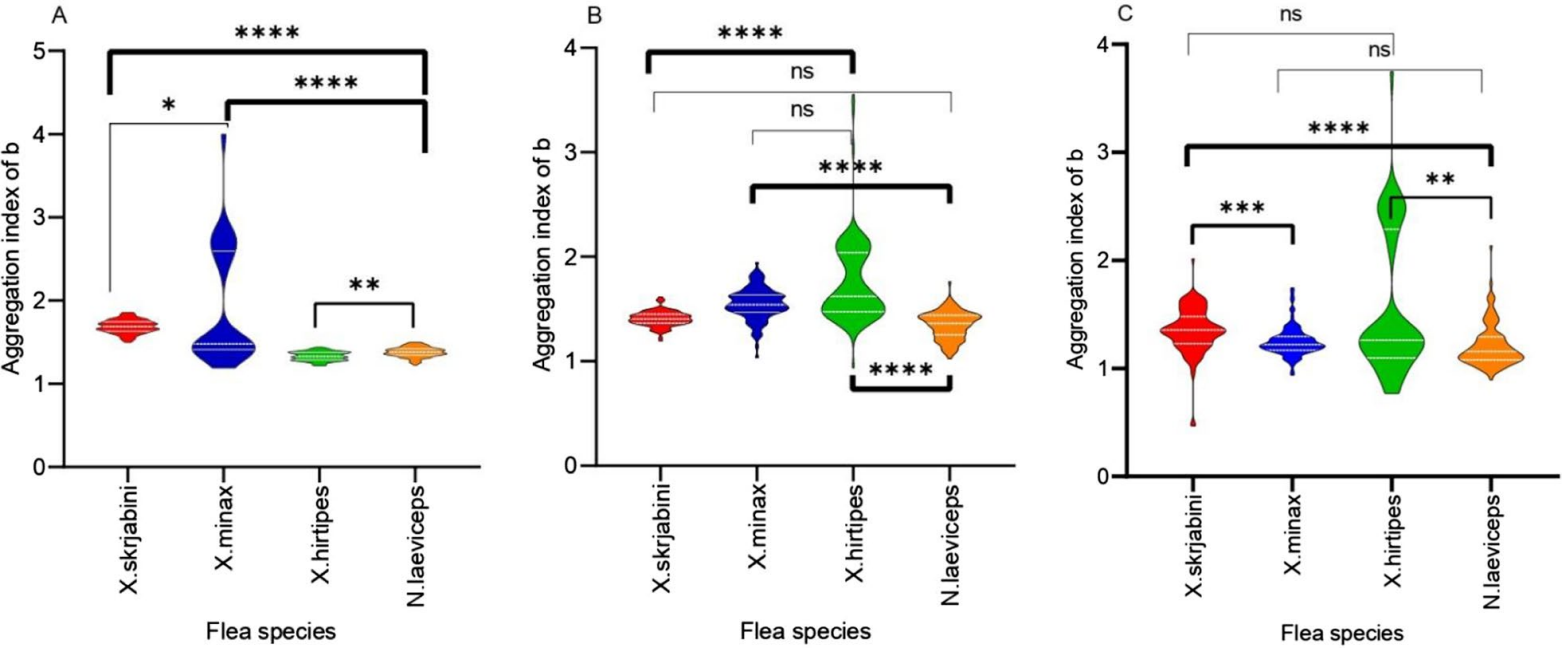
The aggregation levels of primary flea species on great gerbils across varying intensities of plague epidemic regions. **A** The aggregation levels of primary flea species on great gerbils in the eastern zone (III); **B** the aggregation levels of primary flea species on great gerbils in the middle zone (II); **C** the aggregation levels of primary flea species on great gerbils in the western zone (I) *P < 0.05; **P < 0.01; ***P < 0.001; ****P < 0.0001; ns, marginally significant [0.05–0.10]

The aggregation indices of the four primary parasitic flea species exhibited significant variations in the three zones (*H*_I_ = 87.12, *df* = 3, *P*_I_ < 0.001; *H*_II_ = 161.76, *df* = 3, *P*_II_ < 0.001; *H*_III_ = 260.68, *df* = 3, *P*_III_ < 0.001). Moreover, the results of the Dunnett test further revealed a discernible pattern of increasing aggregation degree from the western to the eastern zone of the Junggar Basin. For example, in the western zone (I) of the Junggar Basin, the degree of aggregation between *X. skrjabini* and *X. hirtipes* in the great gerbils did not show interspecific differences (Fig. 2C). Conversely, in the eastern zone (III), the aggregation degrees of the primary parasitic fleas exhibited significant differences (Fig. 2A). Furthermore, the aggregation level of flea species is subject to variation in response to environmental changes and the intensity of plague epidemics. For instance, the degree of aggregation of *X. skrjabini* and *X. hirtipes* in both the eastern zone (III) and the middle zone (II) both showed interspecific differences, but this difference disappeared in the western zone (I) (Fig. 2).

## Discussion

This study aimed to elucidate the aggregation patterns of fleas parasitizing the great gerbils in the Junggar Basin plague natural foci. We identified three primary epidemic areas within this focus, namely the western zone (I), the middle zone (II), and the eastern zone (III), which exhibited variations in flea community structures. Regarding the composition of flea species, although 19 species have been identified, only 4 were predominant in terms of their vectorial capacity, and the composition of dominant fleas varied across zones. In addition, the flea infestation rate exhibited significant differences among the three areas, which was different from the research results of Amatre et al. [23]. Their results did not identify any significant differences in flea infestation rates between villages with or without a history of plague during interepizootic periods; however, our study was conducted during epizootic periods. Furthermore, Wang et al. [19] demonstrated that the flea index of great gerbils was higher in plague epizootics and could be used as an evaluation tool for plague epizootics in this foci. In our study, although there was no significant difference in the total flea index, the dominant flea indices varied among the three zones. In terms of the distribution of the number of fleas per individual great gerbil, our results observed that within the great gerbil population, the majority of individuals carried only a small number of fleas, whereas a minority had a higher aggregation of ectoparasitic fleas, a pattern observed with various other studies [4, 24]. Krasnov et al. [4] further validated this pattern, noting a negative binomial distribution of fleas not only within individual host species populations but also across the entire host community.

Flea co-occurrence is frequently observed in small mammals [12]. Cox et al. [25] explained the phenomenon of concomitant infections by highlighting the impact of parasites on the immune system, particularly through the induction of immunodepression. In our study, we observed that the great gerbils harbored between one and two species of fleas, accounting for 65.6% of the total number of inspected great gerbils. Particularly, the eastern zone (III) exhibited the highest proportion of the flea coinfections, which may be attributed to the highest flea index of *X. skrjabini*. This flea has been confirmed to possess vector capability. When carrying *Y. pestis*, it can weaken the resistance of great gerbils, thereby leading to the high prevalence of flea coinfections in this area. Moreover, in terms of flea abundance, most great gerbils carried between one and five ectoparasitic fleas, accounting for 54.7% of the total number of the great gerbils. However, it was noteworthy that the highest contribution to the total flea index of the great gerbil came from two groups: 6–10 fleas and 11–15 fleas.

Many studies have demonstrated that the distribution of parasites in their hosts populations was highly aggregated [4, 11, 26], and the aggregation affected the stability of the parasite population, the dynamics of transmission, the intensity and prevalence of infection, etc. [7]. At present, a variety of methodologies exist for quantifying the extent of parasite aggregation, including the variance-to-mean ratio, the parameter *k* of the negative binomial distribution, mean crowding, the patchiness index, Poulin’s *D*, Hoover’s index, and Taylor’s power law [12, 21, 27, 28]. Among them, the most commonly used measurement methods are the parameter *k* of the negative binomial distribution and Taylor’s power law [29]. However, the former had its limitations, as it may have underestimated the degree of aggregation within parasite populations when the sample size was reduced, and its parameter *k* may not have accurately reflected the actual level of aggregation of the parasites [30]. In contrast, the parameter *b* of Taylor’s power law was an exponent that was not affected by the size of the sample or the mean of the sample. The value of *b* equal to 1 signified a random distribution, whereas a *b* greater than 1 denoted an aggregated distribution. Conversely, a *b* value less than 1 was indicative of a regular distribution [21]. Taylor’s power law has been found to describe the observed spatial distribution of various organisms, including viruses, plankton, insects, ticks, fish, and other species [20]. Our research results further confirmed that Taylor’s power law was also applicable to explaining the aggregation patterns of fleas parasitizing the great gerbils in the natural plague foci of the Junggar Basin. In our study, the aggregation index *b* varied from 1.20 to 1.82, indicating that the distribution of fleas within the great gerbil population was aggregated. Furthermore, in our study, we observed that the aggregation index *b* of Taylor’s power law was not static [22, 24] but showed variability influenced by both the flea species and the intensity of plague prevalence in distinct zones. We found that the flea aggregation index was significantly lower during the high-intensity epidemic period than during the low-intensity epidemic period. This may be related to the structure of flea communities and the effectiveness of the vectors in the spread of the plague[31]. After areas with low and high rates of plague antibody positivity were compared, it was found that in areas with low plague antibody positivity, the number of species of fleas parasitizing the great gerbil decreased to four, and no combination was found for *X. skrjabini*, which has been proven to be an effective vector for the spread of plague (Additional File 2, Table S2). This finding further confirmed the close relationship between the prevalence of the plague and the aggregation level of its main host animal’s parasitic fleas.

## Conclusions

Consequently, the present study infers three hypotheses regarding the significant role of fleas parasitizing great gerbils in the transmission of plague. Firstly, although the great gerbil was infested with numerous species of fleas, only a few were identified as primary vectors for plague transmission, namely *X. skrjabini*, *X. minax*, and *X. hirtipes*. Experimental evidence has confirmed the vector capability of *X. skrjabini* [32], while field epidemiological investigations have isolated *Y. pestis* from both *X. minax* and *X. hirtipe* [16]. The role of other flea species may serve to facilitate the dissemination of plague within the epidemic area. Secondly, the epidemic of animal plague within the Junggar Basin could be attributed to the high aggregation of its primary vector fleas. These fleas, within a certain period, are concentrated in high numbers on the hosts, thus facilitating the efficient transmission of *Y. pestis* among the host population [33]. For example, *X. skrjabini*, characterized by a short lifespan and explosive population growth, enables it to efficiently transmit *Y. pestis* among populations of great gerbils [32]. Thirdly, in the Junggar Basin plague foci [15], which stretched over 800 km from east to west. The westernmost part of this focus, Alashankou, served as the gateway for Central Asian monsoons that blew from west to east across the Mongolian Plateau. This resulted in a gradual trend of desertification and aridity in the overall climatic microenvironment of the plague focus, moving from west to east. Furthermore, our study revealed that these microclimatic changes had induced variations in the aggregation patterns and levels of the primary fleas in different zones. However, whether such differences lead to varying intensities of plague prevalence in different areas remains to be further explored.

## Supplementary Information

Additional file 1: Table S1. Flea species parasitizing on the great gerbils.

Additional file 2: Table S2. During different periods of parasitological parameters of the primary species of ectoparasitic fleas on the great gerbils in Alashankou within the western zone (I).

## Data Availability

No datasets were generated or analyzed during the current study.
