# Supplementary material for: Study on the aggregation patterns of fleas parasitizing the great gerbil (Rhombomys opimus) in the Junggar Basin plague natural focus
Source: Parasit Vectors. 2025 Feb 13;18:53. doi: 10.1186/s13071-025-06676-4 (PMC11827352; doi:10.1186/s13071-025-06676-4)
Supplement: Supplementary file 1 — Additional file 1: Table S1. Flea species parasitizing on the great gerbils. [file 13071_2025_6676_MOESM1_ESM.pdf]

Table S1. Flea species parasitizing on the great gerbils

Year	No. of sueveys	No. of great gerbils	Flea infestation rate (%)	No. of fleas	Total of flea index	Proportions of individual fleas to the total flea population (%)																		
						X.skrjabin	*X.minax*	*X.hirtipes*	*N.laeviceps*	*C.lamellifer*	*X.conformis*	*N.tersus*	*N.turkmenicus*	*P.teretifrons*	*P.repandus*	*S.meridiana*	*O.kiritschenkoi*	*C.dolichus*	*R.cedestis*	*M.eucta*	*E.oschanini*	*S.conspecta*	*E.tiscadaea*	*S.longispinus*
2005	13	258	77.9	2,032	7.88	25.5	44.4	2.7	13.7	3.0	1.0	0.2	0.0	1.9	5.4	0.4	0.0	0.8	0.7	0.1	0.1	0.0	0.0	0.0
2006	30	794	85.5	5,876	7.38	56.3	7.6	10.2	3.9	6.9	1.8	2.1	0.3	3.4	5.9	0.1	0.0	0.9	0.4	0.0	0.2	0.2	0.0	0.0
2007	3	60	78.3	368	5.82	0.3	81.0	1.6	1.6	0.0	0.5	0.0	5.2	0.0	0.0	0.0	0.0	0.0	0.0	0.0	7.1	0.0	2.7	0.0
2008	4	232	85.8	1,681	7.25	18.9	37.3	6.3	5.0	5.1	0.4	1.7	0.0	9.6	12.6	0.1	0.1	1.4	1.6	0.0	0.1	0.0	0.0	0.1
2009	16	496	84.3	5,373	10.83	61.6	14.4	12..3	5.7	1.3	0.3	0.0	0.0	0.9	0.0	0.0	0.0	0.1	0.2	0.0	3.4	0.0	0.0	0.0
2010	24	418	85.9	2,450	5.86	49.0	14.9	23.0	6.0	2.9	0.0	0.7	0.0	0.0	1.2	0.0	0.0	0.1	0.2	0.0	2.1	0.0	0.0	0.0
Average	14.83	376.33	83.0	2,963.33	7.50	35.3	33.3	9.3	6.0	3.2	0.7	1.0	0.2	2.6	4.2	0.1	0.0	0.5	0.5	0.0	2.2	0.0	0.5	0.0
