# Supplementary material for: Study on the aggregation patterns of fleas parasitizing the great gerbil (Rhombomys opimus) in the Junggar Basin plague natural focus
Source: Parasit Vectors. 2025 Feb 13;18:53. doi: 10.1186/s13071-025-06676-4 (PMC11827352; doi:10.1186/s13071-025-06676-4)
Supplement: Supplementary file 2 — Additional file 2: Table S2. During different periods of parasitological parameters of the primary species of ectoparasitic fleas on the great gerbils in Alashankou within the western zone (I). [file 13071_2025_6676_MOESM2_ESM.pdf]

Table S2. During different periods of parasitological parameters of the primary species of ectoparasitic fleas on the great gerbils in Alashankou within western zone(I).

Year	Periods	No. of the great gerbils	*X.skrjabini*	*X.minax*	*X.hirtipes*	*N.laeviceps*
2005~2008	The high intensity epidemic period	175	0.58	4.83	0.03	0.77
2009~2010	The low intensity epidemic period	108	0.00	7.94	0.01	0.98
